# The adaptive immune system in early life: The shift makes it count

**DOI:** 10.3389/fimmu.2022.1031924

**Published:** 2022-11-17

**Authors:** Daan K. J. Pieren, Mardi C. Boer, Jelle de Wit

**Affiliations:** Centre for Infectious Disease Control, National Institute for Public Health and the Environment (RIVM), Bilthoven, Netherlands

**Keywords:** adaptive immune system, infants, vaccination, respiratory infectious diseases, heterogeneity, tolerance, immune memory, biomarker

## Abstract

Respiratory infectious diseases encountered early in life may result in life-threatening disease in neonates, which is primarily explained by the relatively naive neonatal immune system. Whereas vaccines are not readily available for all infectious diseases, vaccinations have greatly reduced childhood mortality. However, repeated vaccinations are required to reach protective immunity in infants and not all vaccinations are effective at young age. Moreover, protective adaptive immunity elicited by vaccination wanes more rapidly at young age compared to adulthood. The infant adaptive immune system has previously been considered immature but this paradigm has changed during the past years. Recent evidence shows that the early life adaptive immune system is equipped with a strong innate-like effector function to eliminate acute pathogenic threats. These strong innate-like effector capacities are in turn kept in check by a tolerogenic counterpart of the adaptive system that may have evolved to maintain balance and to reduce collateral damage. In this review, we provide insight into these aspects of the early life’s adaptive immune system by addressing recent literature. Moreover, we speculate that this shift from innate-like and tolerogenic adaptive immune features towards formation of immune memory may underlie different efficacy of infant vaccination in these different phases of immune development. Therefore, presence of innate-like and tolerogenic features of the adaptive immune system may be used as a biomarker to improve vaccination strategies against respiratory and other infections in early life.

## Introduction

Respiratory infections within the first year of life are common and can evolve into severe disease. Infectious diseases are indeed a major cause of childhood death. For some infectious diseases causing high mortality at early age, such as invasive infection with respiratory syncytial virus (RSV), *Klebsiella*, and *Staphylococcus aureus*, vaccines are currently unavailable (1–4). Vaccination against respiratory infectious diseases have greatly reduced childhood mortality. Moreover, maternal vaccination has been implemented to circumvent susceptibility to respiratory disease at early age (5) by boosting the number of maternal antibodies that are transferred to the fetus during pregnancy. Implementation of maternal vaccination in several countries (6) has been shown to protect neonates (<1 month old) against respiratory infectious pathogens such as influenza virus and *Bordetella pertussis* that may cause life-threatening disease and may therefore provide the neonatal immune system with the necessary time to evolve into a fully protective immune system. However, achieving early-life immune protection against respiratory infectious diseases for which vaccines are available often requires administration of multiple vaccine doses and even then protection elicited can be of shorter duration compared to later in life, potentially leaving infants at risk for respiratory disease.

The innate immune system is an essential part of the early life immune system as it provides a first line of defense at primary pathogenic encounter. Moreover, already in early life the innate immune system starts to develop a form of nonspecific memory that is trained by pathogen exposure, referred to as trained immunity (7). Trained immunity enhances the response of NK cells and monocytes, amongst other cell types, through encountering pathogens and thereby providing nonspecific protection against a broad range of pathogens. For example, vaccination with Bacillus Calmette-Guérin (BCG) has been shown to induce trained immunity in human adults and infants, possibly providing protection not only against disseminated tuberculosis disease but also against other infectious diseases, as reviewed elsewhere (8). However, in contrast to trained immunity, adaptive immune responses that are led by T cells and B cells are vital to provide pathogen-specific protection by building up long-term memory to specifically prevent or limit disease during following encounters.

The neonatal adaptive immune system has long been regarded immature when compared to the adult immune system. Indeed, infants generate suboptimal (memory) T-cell and B-cell responses after vaccination and against acute infection. However, within both the infant T- and B-cell compartment various cell subsets are present that have inspired a change of perspective on the early life adaptive immune system. The view of immune underdevelopment has changed towards a view of immune tolerance: the immune system at early life starts out at being tolerogenic and then slowly adapts to the new environment to become a controlled inflammatory immune system. Moreover, subsets of the early life adaptive immune system appear to be equipped with unique innate-like features to provide rapid protection.

In this review, we assess current literature to find hallmarks of the unique neonatal adaptive immune system that may help to explain timing of (multiple) vaccine doses in order to boost adaptive immune responses against pathogens early in life. We argue that efficacy of vaccination is determined by the rate at which a neonate shifts from an early adaptive immune system that comprises features of tolerance and innate-like capacities towards a mature system of immune memory. We provide future directions for research to characterize whether the immune response to vaccination may be deemed protective or not.

## T-cell responses at early age

### CD4^+^ helper and effector T cells at early age: rapid innate-like features, low memory generation

The adult adaptive immune system is poised towards providing protection against current pathogenic threats, while also building long-lasting protection by generation of memory cells. A T-cell response is classically initiated by binding of the T-cell receptor (TCR) to its cognate epitope derived from a pathogen that is presented by antigen presenting cells (APCs) (9). Additionally, interaction of co-stimulatory CD80/CD86 on APCs with CD28 on T cells, amongst other co-stimulatory pathways, provides T cells with the necessary second signal that enhances their response. Once these requirements are met, T cells become activated, secrete cytokines, proliferate, and differentiate into specific subsets to provide acute and long-term protection (10). Of note, several APC subsets show differences in infants compared to adults, mainly comprising decreased functionality of dendritic cells, monocytes, macrophages, and granulocytes at young age, as extensively reviewed elsewhere (11). Consequently, altered APC functionality may in turn affect adaptive immune responses.

In adults, naive CD4^+^ T cells that encounter pathogen-derived antigens mainly differentiate into type 1 T-helper cells (Th1) that are characterized by secretion of pro-inflammatory interferon-γ (IFN-γ) and tumor necrosis factor-α (TNF-α) and thereby provide protection against viruses, bacteria, and parasites. However, the CD4^+^ T-cell response to pathogens in neonatal mice and humans has often been reported to be type 2 T-helper cell (Th2)-skewed at the expense of Th1-type responses (12–14). This may in part result in lower levels of Th1-associated cells early in life, whereas the level of Th2-associated cells remains stable with age (15). Th2 responses are characterized by secretion of interleukin (IL-) 4, IL-5, and IL-13 and skewing towards these Th2-type responses may affect vaccine- and infection-related protective T-cell responses. In neonatal mice, antigen recall responses, e.g. elicited by secondary RSV infection (16) or secondary exposure to ovalbumin (14), have been shown to trigger Th2-type responses. However, pioneering studies in neonatal mice have also shown that the shift in balance towards a Th2-type response depends on the type of antigenic stimulation (13, 17). For example, lowering the infectious dose of leukaemia virus resulted in Th1- and cytotoxic T-cell responses in contrast to the non-protective Th2-response with a high viral dose (17). This indicates that the murine neonatal immune system may be poised towards Th2-type responses in case of high infectious dose and/or secondary antigenic encounters.

In human neonates, steady state and polyclonally stimulated CD4^+^ T cells expressed high intracellular levels of IL-4 (18) and polyclonal stimulation also resulted in less IFN-γ production (19) compared to adult CD4^+^ T cells. Moreover, skewing towards Th2-type responses in neonates in vaccination settings has also been reported, although outcomes may vary depending on the type of vaccine and/or adjuvant administered, such as Th2-skewing aluminum-containing adjuvants (20), and subunit versus whole cell vaccines. The recall T-cell memory response elicited after hepatitis B subunit vaccination adjuvanted with aluminum hydroxide was primarily of a Th2-type in newborns in contrast to adults, which possibly also resulted in higher antibody responses compared to adults (21). Moreover, vaccination with a conjugate vaccine against pneumococcus (three doses) adjuvanted with aluminum phosphate in neonates has been associated with Th2-type responses (22), although no adult control group was included. In contrast, generation of robust Th1-type responses has been shown in neonates vaccinated with a BCG vaccine (23) and whole-cell pertussis vaccine (24), indicating that vaccines containing Toll-like receptor (TLR)-agonists may elicit substantial Th1 responses in neonates. In an infection setting, Th2 polarization has been reported in RSV-infected infants with acute bronchiolitis that showed an IL-4 skewed IL-4/IFN-γ ratio compared to infants with upper respiratory tract infection, indicating that increased Th2-type and/or decreased Th1-type responses may contribute to severe disease (25). Th2-skewed responses can be counteracted by the administration of type I IFN-α in mice (26). The role of type I IFN in RSV and infants has been reviewed extensively elsewhere (27).

Interestingly, the naive CD4^+^ T-cell population includes recent thymic emigrants (RTEs) that are highly abundant early in life (15) and express innate-like features such as TLR1 and TLR2 (28–30). Neonatal CD4^+^ T cells produce equal levels of IFN-γ and IL-2 after TLR2-stimulation compared to adult CD4^+^ T cells, whereas TCR stimulation alone induced lower IFN-γ and IL-2 production by neonatal compared to adult CD4^+^ T cells (29). Moreover, neonatal CD4^+^ T cells have been shown to produce the innate effector cytokine IL-8 in response to polyclonal and TLR stimulation (30) in contrast to adult T cells (31). Based on the findings that neonatal T cells adequately respond to TLR stimulation it may be argued that neonatal T cells preferentially use TLRs to recognize pathogens, resulting in cytokine secretion. This in contrast to the classical view of T-cell activation *via* recognition of specific epitopes *via* their TCR (32–34). Innate-like functionality of neonatal CD4^+^ T cells may also explain why some vaccines elicit robust Th1 responses, such as the BCG vaccine, which can trigger several TLRs, including TLR2 (23).

Innate-like effector capacities of neonatal CD4^+^ T cells may indicate that T cells generated at early life differ from those generated later in life. It has indeed been shown that neonatal T cells are derived from different progenitor cells compared to adult T cells, indicating the uniqueness of the neonatal immune system (35–37). Whereas the ‘investment’ of the early life adaptive immune system in innate-like properties may provide protection against acute respiratory pathogenic threats, this investment may also be seen to be at the expense of memory cell formation. As proposed previously (38), the innate-like features of neonatal T cells can be explained by the changing environment: e.g. the vast expansion of the neonatal microbiome makes it difficult for TCRs to distinguish between commensal bacteria and pathogenic bacteria. Also, the capacity of building a stable memory T-cell population is also subject to the process of central tolerance, in which self-reactive T cells get eliminated in the thymus to prevent unwanted responses to self-peptides. The T cells are constantly adapting to discriminate between self-antigens, commensal and pathogenic antigens. The potential to detect harmful pathogenic signals *via* TLRs is therefore highly needed, but at the cost of the capacity to generate a stable memory T-cell population. As a consequence, the downside of investing in innate-like properties may be the diminished response to vaccinations, as well as generation of long-lasting memory response to vaccinations.

Together, these findings indicate that I.) the neonatal T-cell response is not necessarily impaired, but may intrinsically be wired towards induction of Th2 responses during TCR-mediated responses, and II.) aside from Th1/Th2 skewing, the CD4^+^ T-cell compartment heavily ‘invests’ in innate-like T-cell features for rapid effector responses and immediate protection at the time when the capacity of memory formation is still developing, thereby compromising long-term protection.

### CD8^+^ effector T cells

Effector CD8^+^ T cells are essential for short and long-term protection against respiratory and other infectious diseases following infection or vaccination. A major part of the T-cell population of neonates consists of RTEs (39), which functionally and epigenetically differ from naive T cells that have matured by circulating for a longer time (40, 41). Neonatal CD8^+^ T cells have been shown to express innate-associated molecules TLR2 and TLR5 (42). Moreover, neonatal murine CD8^+^ T cells have been shown to express NK cell-related transcripts and molecules (36, 43, 44) in contrast to adult CD8^+^ T cells, which again supports the innate-like features of neonatal T cells.

In addition to these innate features, the transcriptional profile of neonatal CD8^+^ T cells has been shown to be enriched for genes associated with cell cycle and anti-viral innate immune responses compared to adult CD8^+^ T cells, potentially to compensate for reduced levels of genes associated with cytotoxic T-cell function (45). Enrichment for genes associated with the cell cycle translated into higher proliferative capacity of neonatal CD8^+^ T cells compared to adult CD8^+^ T cells (45). The capacity of murine neonatal CD8^+^ T cells to rapidly expand after stimulation has been shown to result in a pool of short-lived effector cells that became terminally differentiated, whereas stimulation of adult CD8^+^ T cells resulted in a diverse pool of effector and memory T cells (46). A more recent study indeed showed that CD8^+^ T cells generated early in life show a more effector-like phenotype before stimulation and respond more rapidly after stimulation, whereas adult-derived CD8^+^ T cells comprise a pool of naive CD8^+^ T cells that are capable of forming a stable memory population (36). The difference in generation of CD8^+^ T cells between neonates and adults in response to infection is that CD8^+^ T cells of neonates may be derived from a different hematopoietic stem cell lineage (43). Lastly, the decay rate of CD8^+^ T cells produced at young age is high, which slows down with progressing age (47), indicating rapid turnover of these cells.

Together, it appears that also the neonatal CD8^+^ T-cell population is poised towards rapid effector and proliferation mechanisms to combat acute potentially harmful pathogens, whereas the capacity to generate memory CD8^+^ T cells is still developing, which poses a window for potentially life-threatening infections to occur. Moreover, reduced memory generation may also limit long-term vaccine efficacy. CD8^+^ T-cell proliferation to measles antigens after vaccination are lower in infants (6-12 months of age) compared to those of adults (48). Although these findings do not mean that the CD8^+^ T-cell response of these infants is not enough to provide protection against measles virus infection, it does indicate that not only neonates but also infants still show altered T-cell responsiveness, which may open a window to infection and/or decreased vaccine efficacy.

### Unconventional T cells: γδ T cells and MAIT cells

The group of unconventional T cells are comprised of several subsets of cells, including T cells expressing the γδ T-cell receptor (γδ T cells) and mucosal associated invariant T cells (MAIT cells). γδ T cells bridge the innate and adaptive features of the immune system due to their capacity to act as antigen-presenting cells (49) and their activation is not restricted to MHC (50). It is currently thought that γδ T cells are one of the main lines of defense against pathogens during early life, at the time when protection mediated by conventional αβ T cells is still developing (50, 51). Indeed, a recent study has shown that γδ T cells rapidly expand and functionally develop in preterm and term infants in a short period of time, whereas αβ T cells show little development (52). This again illustrates a possible investment in innate-like T-cell features by the neonatal immune system. T cells expressing the γ-chain variable region 9 (Vγ9) and δ-chain variable region 2 (Vδ2) are most abundant in human peripheral blood, already providing an innate-like T-cell barrier in the developing fetus (53). It has been suggested that γδ T cells are a main line of defense against pathogens during cytomegalovirus infection already *in utero* (54), and γδ T cells were shown to produce IFN-γ after BCG vaccination in infants (55). Moreover, a recent study shows that shortly after birth (<10 weeks) Vγ9Vδ2 T cells differentiate into cytotoxic effector cells expressing granzymes and perforin similar to adult levels (56). However, their IFN-γ response to bacterial antigens such as the microbial-derived (E)‐4‐hydroxy‐3‐methyl‐but‐2‐enyl pyrophosphate (HMBPP) was lower compared to adults. Indeed, a recent study shows that the a γδ T-cell compartment is not completely established in neonates 14 days after birth, as γδ T cells of older children show higher IFN-γ responses to bacterial-derived antigens (57). Together, these studies indicate the importance of γδ T cells as a first line of defense against infections at early life, highlighting the importance of innate-like functionality at early age.

MAIT cells are a subset of T cells already present in neonatal cord blood that recognize vitamin B metabolites presented by MR1, which is an MHC class I-related protein (58). MAIT cells have been found to recognize cells infected with *Mycobacterium tuberculosis* (59), a pathogen which may cause severe disease in infants. Similar to γδ T cells, it has recently been shown that the population of MAIT cells rapidly expands and matures after birth (60, 61). Moreover, the functional TNF response of infant MAIT cells to *Mycobacterium smegmatis* was found to be greater than the response of adult MAIT cells (60). Lastly, the precise role of MAIT cells in vaccination remains to be established, as BCG vaccination in infants did not alter MAIT cell activation or memory phenotype (62), whereas MAIT cells have recently been shown to enhance the efficacy of an adenoviral vector COVID-19 vaccine (ChAdOx1) in humans by improving CD8^+^ T-cell responses (63). Together, these studies indicate that MAIT cells may play an important role in prevention of infections early in life.

## Tolerance by T cells at early age

### CD4^+^ Regulatory T cells

Immune tolerance is needed to prevent inflammatory innate-like immune responses during gestation (64) and to allow establishment of the microbiome after birth. As described above, neonatal T cells have innate-like effector capacities for rapid effector function, whereas the capacity to generate immune memory is still developing. As a consequence of these innate-like effector capacities, a strong, tightly regulated tolerogenic system is needed to dampen excessive inflammatory responses and to thereby prevent collateral immune-mediated damage.

During pregnancy, immune tolerance of the mother towards the fetus ensures fetal growth and development. Maternal tolerogenic M2-like macrophages, natural killer cells (NK cells), and regulatory T cells (Tregs) accumulate within the decidual wall to provide tolerance in order to prevent fetal rejection, but also to limit pathology during infections (65, 66). Similarly, fetal suppressive CD4^+^ CD25^+^ Tregs already appear in fetal tissue from 13 weeks of gestational age (67), and these fetal Tregs have been show to play an important role in immune suppression of CD4^+^ and CD8^+^ T-cell proliferation and cytokine secretion already in the absence of stimulation (35, 68).

The level of Tregs in neonatal cord blood and peripheral blood compared to adult peripheral blood has been shown to be higher (69–72). Moreover, newborn nonhuman primates also show higher levels of Tregs in the spleen and lungs in addition to peripheral blood (73), suggesting that increased presence of Tregs outside of the blood may also occur in human infants. However, a slight increase of Tregs with age when comparing cord blood with (young) adult peripheral blood has also been reported (74). A reason for these contrasting results is still unknown, although a difference in identification of Tregs by phenotypic markers, as well as definition of Treg subsets may account for these differences. Moreover, it may well be that the pace at which the shift from higher levels of Tregs in the fetus/neonate towards adult levels of Tregs occurs, differs per individual. It is tempting to speculate that this heterogeneity in tolerogenic shift on the individual level could subsequently determine the outcome of immune responses towards infection and vaccination. Thus, it may be valuable to assess Treg levels longitudinally on the individual level to determine whether some infants shift less rapidly than others. This may give an answer to whether heterogeneity in conversion from tolerance to inflammatory and memory formation is a proxy for response to vaccination and/or susceptibility to infection.

Reports on the functionality of neonatal Tregs are contrasting, but may be explained by the different experimental approaches, e.g. cord blood versus peripheral blood, as well as the cell type that is used as a readout in suppression culture assays. Cord blood-derived CD127^Lo^ CD25^+^ Tregs were found to suppress a mix of CD4^+^CD25^-^ T cells and dendritic cells as potently as the most suppressive (CD25^Hi^) adult-derived Tregs (75), or suppress CD4^+^CD127^Hi^CD25^-^ cells to the same extent as adult Tregs (74). Other studies report ambivalent results: expanded cord blood-derived CD25^+^ Tregs suppressed CD4^+^CD25^-^ T cells to a greater level than adult peripheral blood-derived Tregs (76), or, in contrast, report that cord blood-derived CD127^Lo^ CD25^+^ neonatal Tregs are less functional in suppressing dendritic cells compared to adult Tregs (77). Finally, a recent study showed that CD127^Lo^ CD25^+^ Tregs in peripheral blood of adults and term neonates suppressed proliferation of CD4^+^CD25^-^ T cells to the same extent, but that both suppressed these cells to a lesser extent than Tregs of preterm neonates (78), which seems to suggest that the suppressive potential of Tregs seems at its peak during gestation and declines after birth, together with declining Treg numbers.

Possibly, the pace at which the shift from high suppressive capacity to modest suppressive capacity occurs, influences susceptibility to respiratory infection. Presence of FoxP3^+^ Tregs during viral respiratory infections such as RSV has been shown to limit RSV-induced pathology caused by eosinophilia, CD4^+^ and CD8^+^ T cells in wildtype and FoxP3 DTR mice (allowing depletion of FoxP3^+^ Tregs) (79–81). The importance of Tregs in infants with severe RSV disease has also been shown, as reduced levels of activated Tregs were demonstrated in severe RSV infection (82), which may be needed to counteract pro-inflammatory IL-17-producing T cells (83). Thus, Tregs are important to dampen harmful inflammatory responses. However, this tolerogenic environment may also dampen inflammatory responses to vaccinations, which could be essential to elicit optimal differentiation of the adaptive responses, required for long term protection. Studies investigating the potential suppressive role of Tregs during vaccination against infectious diseases are relatively scarce. In infants, BCG vaccination was shown to induce expression of FoxP3, indicating Treg induction (84). Of these infants, some showed IFN-γ responses whereas other infants showed IL-10 responses to mycobacterial purified protein derivative, indicating immunological variation within the group of infants. Another study indeed suggests that CD25^+^ Tregs are involved in IL-10 secretion in response to BCG vaccination of neonates (85). In infants vaccinated against measles and Diphteria/Tetanus/Pertussis (DTP), a (relatively weak) negative association between the number of CD127^Lo^ FoxP3^+^ Tregs present in the periphery and the antibody response to measles vaccination, but not DTP vaccination (Diph-Tet-Pert) was shown (86). Together these data indicate a potential suppressive role for Tregs during infant vaccination, although this may depend on individual Treg variation as well as the type of vaccine.

As neonatal T cells show innate-like characteristics, Tregs may have to deal with these innate-like capacities and respond to innate signals to prevent excessive inflammation. It has been shown that murine and human CD25^+^ and/or FoxP3^+^ Tregs express several TLRs (87–90). Exposure to TLR4 and TLR5 ligands, such as lipopolysaccharide (LPS) and flagellin, induces Treg suppressive activity and survival in the absence of TCR-mediated activation (87, 88, 90). TLR2 activation on the other hand, directly promotes Treg proliferation, but limits their suppressive capacity (89, 91–93), indicating that this innate feature is a mechanism to bypass the intrinsically tolerogenic wired neonatal immune system.

### CD8^+^ regulatory T cells

CD8^+^ Tregs may also be involved in suppression of neonatal T-cell responses. For example, it has been shown that an important role of Qa-1-restricted (HLA-E in humans) CD8^+^ Tregs is to prevent autoimmune disease in adult mice by suppression of follicular helper T cells (94). Moreover, genetic disruption of these CD8^+^ Tregs resulted in increased clearance of acute and chronic viral infections in mouse models (95). However, studies addressing the role of CD8^+^ Tregs in human infants and neonatal mice are scarce. One study in a transplantation model of neonatal mice showed that long-lived CD8^+^ Tregs could prevent graft rejection by CD8^+^ T cells and these Tregs maintained tolerance at later life (96). Presence of CD8^+^ Tregs in human infants requires to be addressed, as this cell type could provide more insight into susceptibility to respiratory infectious disease and vaccination responsiveness.

### Virtual memory T cells

Virtual memory CD8^+^ T cells (TVM cells) were first identified in unimmunized wildtype and germ-free mice and were described as cells that express a memory phenotype without having been exposed to antigen (97). Human TVM cells are characterized by expression of CD45RA and innate-like receptors NKG2A and several KIRs (98). TVM cells most likely develop *via* cytokine-induced homeostatic proliferation (97). In neonatal mice, TVM cells have been shown to be present in the periphery from two weeks of age, and their expansion was partly dependent on IL-4 (99). As CD4^+^ T cells of humans and mice show Th2-skewing towards IL-4 at early age, it may well be that the initial expansion of TVM cells is elicited by IL-4 secreted by Th2 cells.

The role of TVM cells in humans and mice is still being elucidated. TVM cells in mice have been shown to provide bystander protection during *Listeria monocytogenes* infection (100). Moreover, TVM cells have been shown to contribute to the control of HIV-infected CD4^+^ T cells in humans, likely through KIR-mediated interactions and killing of infected cells (101). Furthermore, our group has recently shown that KIR-expressing TVM cells accumulate in the blood with progressing age and are capable of suppressing proliferation of other CD8^+^ T cells (102). Based on these findings, TVM cells may also be considered a type of suppressive CD8^+^ T-cell subset, although their role at early age remains to be elucidated. Moreover, it has been shown previously that CD8^+^ Tregs expressing the innate receptor NKG2A can recognize HLA-E in an antigen specific manner and can both exert killing as well as regulatory suppressive functions (103, 104), which suggests a link between TVM cells and these HLA-E restricted CD8^+^ T cells. It would be of interest to address TVM cells in human neonates and infants as these cells may possibly be another feature of the adaptive early age immune system to invest in innate-like T-cell features and with potentially both pathogen-clearing and suppressive capacities.

### Immune regulation by co-inhibitory receptors on neonatal T cells

The number of studies addressing expression of co-inhibitory receptors by neonatal T cells is limited. Expression of inhibitory receptors LAIR-1, CD31, and CD200 was increased on neonatal T cells derived from cord blood and peripheral blood compared to adult peripheral blood (105), indicating a potential mechanism for regulation of responsiveness of these T cells. On the other hand, these data could also indicate that excessive T-cell effector capacities are dampened to prevent pathology. Recently, expression of the C-type lectin CD161 by CD4^+^ T cells was shown to inhibit TCR-dependent induction of IFN-γ and these cells were present at a higher level in cord blood of newborns with chronic inflammation of the intestines as a consequence of gastroschisis (44). Together, expression of co-inhibitory receptors by infant T cells remains a relatively unexplored field which may provide new leads for investigation of reduced vaccine responsiveness.

**Figure 1:**
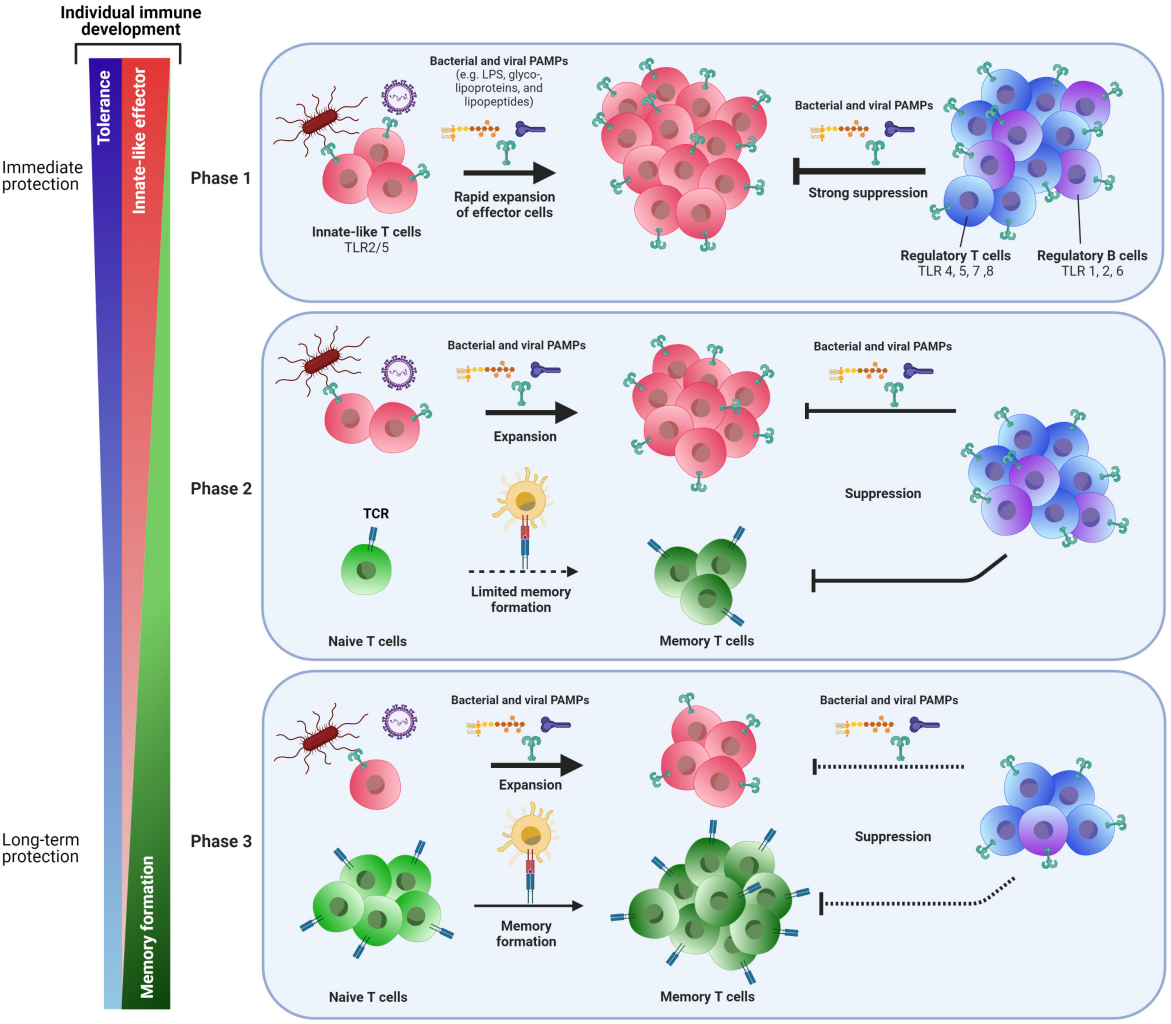
Phasic shift in the adaptive immune response to pathogens early in life: from immediate to long-term protection. Cells of the adaptive immune system change during the course of life. At birth (Phase 1), neonatal T cells express innate-like features such as TLRs 2 and 5, allowing these cells to recognize bacterial and viral PAMPs and act upon pathogenic threats by rapid expansion of these innate-like T cells and by exerting effector functions. To prevent immunopathology, a vast population of regulatory T and B cells can also recognize pathogens *via* several TLRs and exert suppressive responses to keep innate-like T-cell responses under control. Together, these mechanisms may provide short-term protection against pathogens, but does this at the expense of formation of memory T cells for long-term protection. Moreover, the efficacy of vaccines that do not trigger TLRs may be reduced early in life. With progressing age, the capacity to generate a population of memory T cells through TCR-MHC interactions of naive T cells with antigen presenting cells slowly develops (Phase 2). Innate-like T cells are still present at this stage, as well as suppressive regulatory T and B cells. Finally (Phase 3), formation of memory cells becomes the main pool of cells that confers protection to pathogenic threats and allows for long-lasting vaccine-mediated protection. Suppressive regulatory T and B cells are still present and needed to maintain the immunological balance. Whereas the shift from phase one to phase three may roughly be put alongside aging over time, there still is individual variation at which these changes to immune cells occur. Possibly, the pace at which the early life’s immune system shifts from acute innate-like and tolerogenic responsiveness towards memory generating responses for long term survival may determine whether some infants will be more rapidly protected, while others may be prone to more severe disease and/or reduced vaccination responses.

Collectively on T-cell responses at early age: whilst innate-like and tolerogenic features have developed and as T-cell memory generation is low at early age, we speculate that the shift from innate-like features to generation of immune memory is an important step in the process to confer protection in childhood and beyond (**Figure 1**).

## B-cell responses at early age

### Generation and maintenance of protective antibody levels in neonates and infants

Next to T-cell immunity, the adaptive immune response that consists of B cells producing antibodies, seems also be differently regulated at early age. Vaccine- and infection-induced antibody responses in neonates are at a lower level and lower in affinity (106–112). Moreover, antibodies generated in early life wane rapidly, with low or undetectable levels at 6-9 months after primary and booster immunization against diphtheria and pertussis between 2 and 6 months of age (113, 114). It is likely to assume that here suboptimal priming of B cells takes place at an early age, inducing short-lived plasma cells. The insufficient vaccine efficacy by rapid waning of antibody levels in infants may decreased protection to disease.

Whereas extremely premature born neonates (<28 weeks of gestational age) were recently shown to be capable of mounting IgG antibodies against most antigens present in DTaP-IPV-Hib-HepB vaccines comparable to term born neonates (115), generation of protective antibodies is still not optimal at young age. Although neutralizing antibodies elicited by maternal vaccination provide protection for 3-4 months after birth, the levels of these antibodies decline over time (116). For measles, vaccination generally starts at 12 months of age, which leaves a window of opportunity for infection to establish as the capacity for long-lived antibody production in neonates starts to increase gradually from 6 to 9 months of age (107, 117). Earlier vaccination of infants may be a solution to narrow this window for infection. However, recent studies show that earlier vaccination against measles results in a long-term decrease in concentration and avidity of neutralizing antibodies below the cutoff for clinical protection against measles (118), as well as a rapid decay of their polyfunctionality (119). Together, these studies indicate that not only generation of antibodies is reduced early in life, but also the maintenance of antibody levels, which may be caused by less sustained maturation and differentiation of memory B cells and plasma cells at young age, resulting in suboptimal protection later in life.

### Follicular helper T cells

Generation of protective antibodies requires a chain of events to occur to elicit robust, protective, and long-lasting antibody responses. Follicular helper T cells (TFH cells) bridge the interaction between T- and B-cell populations (120). TFH cells mediate generation and maintenance of B-cell germinal centers (GCs) where affinity maturation takes place and B-cell differentiation towards long-lived memory B cells and plasma cells producing high-affinity class-switched antibodies (121, 122).

Functional TFH cells are characterized by several factors, including expression of the transcriptional master regulator Bcl-6, as well as IL-21, and IL-4 (120, 121). Impaired TFH cell generation and differentiation may subsequently affect generation of protective B-cell responses. In neonatal mice, vaccine-induced TFH-cell generation is impaired, as characterized by lower expression of Bcl-6 and IL-21, as well as reduced migration into germinal centers, which may be the cause for reduced antibody responses found in these mice (123). Similarly to neonatal CD8^+^ T cells (45), neonatal mouse TFH cells were shown to be enriched for cell cycle-related genes, indicating that also this T-cell subset in neonates is poised towards rapid action instead of memory generation (124). Moreover, TFH cells elicited in neonatal mice showed a bias to Th2 cytokines, which may negatively affect further generation of TFH cells (124). Adjuvantation of vaccines with CpG oligodeoxynucleotide (CpG-ODN), amongst others, have been shown to reduce Th2 bias and thereby overcome reduced TFH-cell and antibody responses, indicating that providing the right stimulus can result in more optimal responses (125, 126). Whereas circulating CXCR5^+^ Tfh cells in humans are important for generation of B-cell memory against influenza (127), there currently are, to the best of our knowledge, no studies that address circulating TFH cells at early life. Thus, strong TFH responses are crucial for developing robust antibody and (memory) B-cell responses. In neonates, reduced TFH-cell responses may affect the remaining chain of events that would normally lead to robust B-cell memory and protective antibody responses.

### Suppression of antibody production by TFH cells through IL-10

Both IL-21 and IL-6 are important for TFH differentiation and therefore contribute to boosting of antibody responses and B-cell differentiation (121, 128). Whereas IL-21 provides signals to promote lymphocyte differentiation (129) and has been implicated in the development of several autoimmune diseases (130), IL-21 can paradoxically elicit immunosuppressive responses through induction of IL-10 secretion by T cells. Interestingly, polyclonal stimulation of naive CD4^+^ T cells from cord blood with anti-CD3, anti-CD28, and IL-21 results in more IL-10 production compared to adult naive CD4^+^ T cells and thereby suppressed proliferation of CD4^+^ T cells (131). More evidence for TFH/IL-10-mediated suppression may be found from studies investigating the other spectrum of age: old age. At older age it is well known that levels of both pro- and anti-inflammatory cytokine levels increase, one of which is pro-inflammatory IL-6 (132). A recent study has shown a pivotal role for the balance between IL-6, IL-10, and IL-21 in the generation of so-called TFH10 cells in aged mice (133). These TFH10 cells derived their name from their capacity to produce IL-10 which is propagated by production of IL-21 to act as a positive feedback loop for TFH10 generation and maintenance. IL-10 secretion subsequently limited antibody responses to influenza vaccination and the authors speculate that this IL-10 production is established to compensate for the pro-inflammatory micro-environment created by IL-6 (133). Possibly, these findings suggest that neonatal IL-10-producing TFH cells may contribute to decreased B-cell responses and antibody levels found at young age. If IL-10-producing TFH cells are indeed involved in suppressing neonatal B-cell and antibody responses, it would be of high interest to address whether these cells become less tolerogenic as age progresses. Together, they may provide new leads to susceptibility to respiratory infectious disease and reduce vaccine responses early in life.

### B cells: Class-switch recombination and somatic hypermutation

As noted above, an impairment in the chain of events that lead to generation of high-affinity antibodies may affect short- and long-term antibody generation and maintenance. B cells of both term and preterm born neonates have been shown to express lower levels of several co-stimulatory receptors, including CD40, CD80, CD86, and some TNFR family receptors (134), which may impede their cross-talk with T cells. Indeed, activation of neonatal B cells with CD40L resulted in lower IgG and IgA production compared to adult B cells (134).

Once antigenic exposure has taken place, interaction with T cells is required for B cells to undergo class-switch recombination (CSR), recently shown to occur before GC formation (135). Subsequent GC formation is required to propagate B-cells and generate high affinity antibody responses. However, impaired formation of GCs and lower presence of TFH cells within GCs of neonatal mice may result in lower antibody generation (123), and may also affect generation of long-lived B-cell memory and plasma cells and thus long-term antibody levels. CSR of B cells is followed by T-cell independent affinity maturation *via* somatic hypermutation (SHM) of the B cell receptor within the GC. It has recently been shown that B-cell SHM increases during the first three years of life (reaching 60-75% of adult SHM frequencies), mainly due to increasing antigenic exposure (136), indicating that B cells of neonates may not yet be sufficiently capable of generating higher-affinity antibodies.

It has recently been shown that numbers of total memory B cells and plasma cells are low in peripheral blood of newborns, but the number of these cells rapidly increases within 11 months of age, reaching their maximum number at approximately 1 year of age (137). However, these are total levels and may not reflect vaccine-induced responses. Whereas vaccination against meningococcal serogroup C was shown to be effective in producing antibodies in infants after two doses instead of three doses (138), another study showed that multiple doses of a conjugated MenC vaccine were required to induce memory B cells and plasma cells (139), showing suboptimal induction of B-cell response after the first vaccination. This indicates that the short-term induction of antibodies may be adequate in some vaccines, but long-term protection mediated by memory B cells and plasma cells may be suboptimal at early age.

### Regulatory B cells (Bregs)

Regulatory B cells (Bregs) secrete high levels of IL-10, transforming growth factor-β (TGF-β), and IL-35 and are thereby capable of suppressing other cell types (140, 141), including various T-cell subsets, as well as the induction of Tregs (142). Differentiation of immature B cells into IL-10-producing Bregs has been shown to be driven by IFN-α secreting plasmacytoid dendritic cells (143). Interestingly, skewing to Th2-type responses in neonatal mice may be explained by IL-10-producing Breg that can be triggered *via* TLR2, TLR4, and TLR9 (144, 145) as secretion of IL-10 by B cells limits dendritic cell-mediated priming of Th1-type responses (144). There is only a limited number of studies addressing the role of Bregs in human infection and vaccination, especially at early age. The frequency of cord blood Bregs within the B-cell population is higher compared to adult peripheral blood and these Bregs inhibit IFN-γ (Th1) and IL-4 (Th2) responses (146), suggesting that these cells play a role in the Th1/Th2 balance at young age (146). Similarly, the frequency of neonatal Bregs (nBregs) amongst the B-cell population is higher in peripheral blood shortly after birth (1-4weeks) compared to one year after birth and adulthood (147). Infants that suffer from severe RSV infection show RSV-infected nBregs that produce IL-10, which in turn affected protective Th1 responses (147). The frequency of nBregs present in the blood positively correlated with symptom severity, indicating the negative effect of suppression. Interestingly, nBregs have also been addressed as being part of an innate-like B-cell population (148), which indeed produce IL-10 after type I IFN secretion induced by adjuvant-mediated TLR9 stimulation (149). Together, these findings show that innate-like and regulatory cellular characteristics are not only present within the T-cell population, but can also be found within the B-cell population at early life.

### Antibody production: Interference by maternal antibodies

It is well known that maternal antibodies provide protection shortly after birth to protect the infant against harmful pathogenic respiratory infections (150–153). Moreover, maternal immunization is now being used as a way to increase protection of the newborn, as extensively reviewed elsewhere (154). Additionally, maternal vaccination may potentially be an important strategy to provide time for the newborn’s immune system to switch from a tolerogenic/innate-like adaptive immune system towards a well-balanced memory generating immune system. Possibly, neonates and infants who are ‘slow switchers’ may especially benefit from maternal vaccination, as it may provide more time to make the shift. However, maternal antibodies (MatAbs) have also been shown to blunt infant immune responses (155–157), which may be an additional explanation for reduced antibody generation in early life. Indeed, a recent study has shown that maternal tetanus, diphteria, and acellular pertussis (Tdap) vaccination elicits MatAbs that interfere with primary and booster vaccination antibody concentrations in newborns (158). As studies have shown that MatAbs can negatively interfere with the neonatal response to vaccines, MatAbs may thus be another hurdle to overcome for the neonate to provide itself with adequate protection. The mechanism behind inhibition of early-life antibody responses by MatAbs remains under debate. MatAbs can interfere with the immune response by binding vaccine-derived immunodominant epitopes, which leaves only non-immunodominant epitopes to be bound by neonatal B cells. This may result in hampered B-cell differentiation and weak antibody responses. A recent study in mice provides evidence that vaccine-induced high levels of MatAbs may also impair expansion of TFH cells and germinal center B cells (159). This study shows that MatAbs impair differentiation of neonatal B cells towards plasma cells and memory B cells, and also alters expression of the B-cell receptor by B cells and thereby the B-cell repertoire (159). Thus, this indicates that future research may search for the fine line between eliciting enough MatAbs through maternal vaccination to protect the neonate, but not in such high levels that MatAbs impede antibody generation by the neonatal immune system. The effect of MatAbs on human B-cell and T-cell differentiation remains to be further investigated.

### Heterogeneity in the pace of adaptive-immune shifting from innate-like to long-term memory

**Figure 2:**
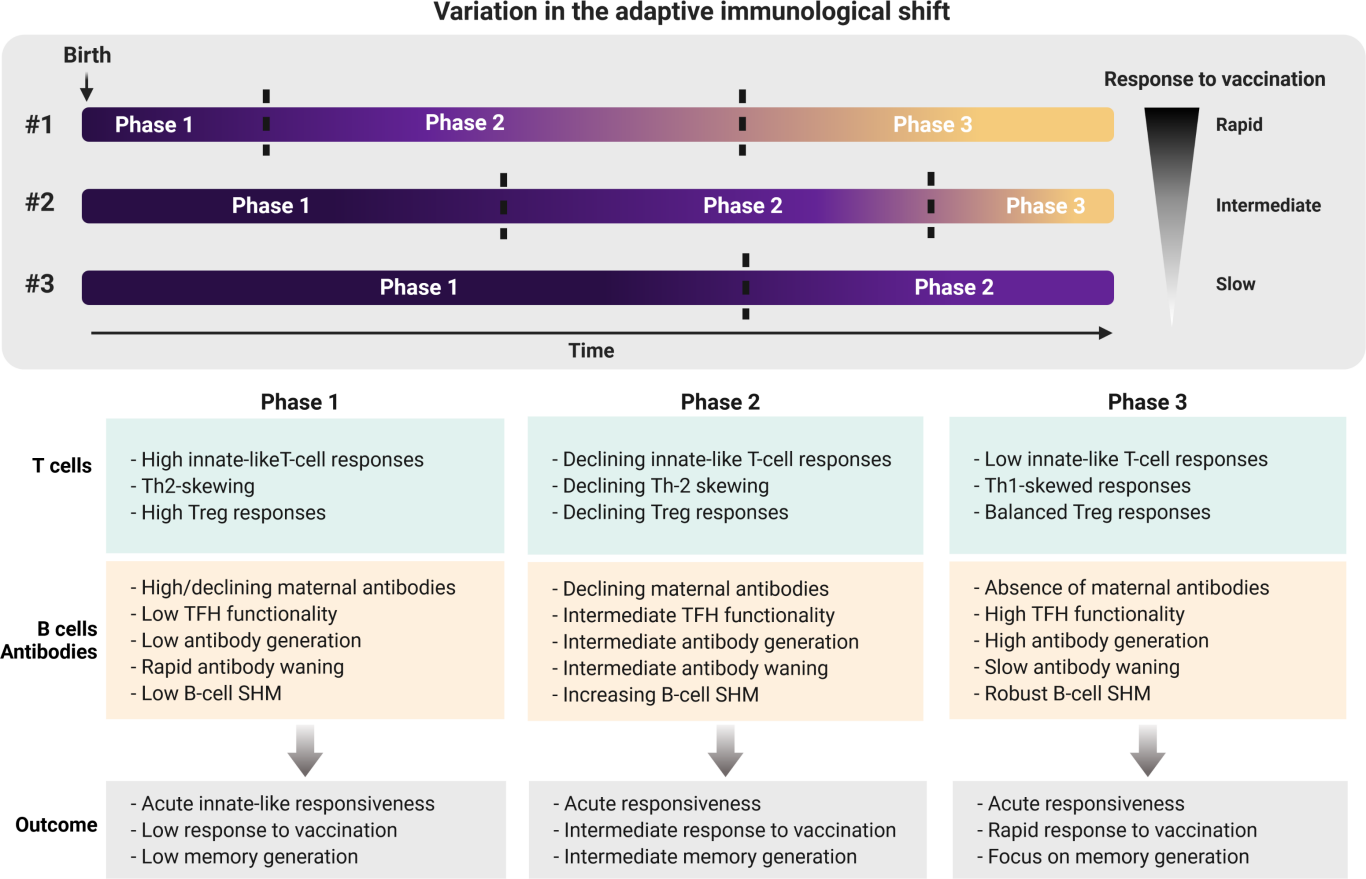
Individual variation in shifting from adaptive innate-like and tolerogenic features early in life towards memory generation later in life. The pace at which the adaptive immune system of newborns shifts from a system with high innate-like and tolerogenic features towards an adult-like memory generating system may determine the susceptibility to infectious disease, as well as the protective efficacy of vaccinations. Neonates who are ‘rapid shifters’ (#1) possibly are better at generating immune memory as a result of infection and/or vaccination for long-lasting protection compared to ‘intermediate shifters’ (#2) and ‘slow shifters’ (#3). Future studies addressing hallmarks of shifting through these phases early in life may therefore provide new insights into whether neonates or infants require multiple doses of vaccinations or not. Th, Thelper; Treg, regulatory T cell; TFH, follicular helper T cell; SHM, somatic hypermutation.

Collectively, published evidence shows a previously less recognized role for the shift from an innate-like and tolerogenic-orientated immune system towards a system of memory formation early in life. We speculate that the shift from innate-like features to generation of immune memory is an important step in the process to confer protection in childhood and beyond (**Figure 1**). However, the speed at which this process takes place may vary between individuals of the same age. Multiple heritable and non-heritable influences may have an impact on the pace of this immunological shift, as indicated by several reports on the variation within the human immune system (15, 160–162). For example, the frequency of TLR-expressing RTEs amongst naive CD4^+^ T cells varies between ∼10-80% early in life, declining thereafter (15). Based on these reports, we propose that the pace at which a newborn child shifts from its innate-like and tolerogenic adaptive immune features towards formation of immune memory may determine the efficacy of infant vaccination and/or their susceptibility to respiratory infection (**Figure 2**).

A major question that remains is by what factors the pace of the early life’s immunological shift is driven. Identifying these factors in future research will be challenging, as it most likely will be a multifactorial process. First, immune development over time is an important factor. Many adaptive immune parameters change early in life, including those in the B-cell (137) and T-cell populations (163). Duration of the pregnancy may be an important factor in this, as immune development consists of several layers, first establishing mutual tolerance to prevent damaging alloreactions, and then slowly evolving into functional effector immune capacities (64). Possibly, the speed of this evolvement is a heterogeneous process, and as infants are vaccinated chronologically after birth, and preterm neonates may therefore have a different immune composition compared to term neonates (164). Second, the (microbial) environment may influence the shift, due to the vast perinatal and postnatal exposure to a novel microbiome (38). For example, perinatal inflammation may alter immune development (52), and the mode of delivery determines the composition of the neonatal microbiome and associated with respiratory infections in the first year of life (165). Another environmental example may be the classical hygiene hypothesis, as exposure to pathogens or antibiotics early in life may determine the nature of consecutive immune responses (166, 167). A way to address potential immune modulatory effects may be through administration of a BCG vaccination (168). Lastly, genetics may play a role in the pace of adaptive-immune shifting. As the early life adaptive immune system relies on innate-like characteristics, genetic variation in TLRs (169, 170) may influence the response to vaccination and infection (171). Thus, the pace at which the adaptive immunological shift at early age may be subjected to heritable and non-heritable factors, and we hypothesize that the phase at which a neonate or infant resides in may determine the effectiveness of vaccination and/or the level of protection elicited against infectious pathogens.

## Conclusion and future perspectives

Susceptibility to respiratory infectious diseases and reduced response to vaccination in neonates can be ascribed to a differently wired adaptive immune system. The neonatal immune system must be aggressive enough to clear pathogens to ensure survival of the newborn, whereas it should also allow the microbiome to establish. Focusing on comparisons between neonatal adaptive immune capacities by comparing these to those of adults may be a one-dimensional view, and is secondary to the fact that the neonatal adaptive immune system is wired differently: it has characteristic innate-like and tolerogenic features to combat pathogens. The neonatal adaptive immune system should therefore be recognized as a system that can respond to acute pathogenic threats by rapid innate-like effector responsiveness. Concomitantly, this rapid innate-responsiveness is tightly controlled by tolerogenic cells to prevent collateral damage, whilst also allowing the neonatal immune system to invest in more time-consuming processes, such as the generation of naive T cells and the capacity to shift to features that promote memory differentiation. The presence of MatAbs provides protection during the first months after birth and thereby provides the time necessary to shift towards immune memory formation. However, interindividual variation in the development of the adaptive immune system may dictate the speed of this immunological shift and we argue that this may determine the effectiveness of vaccination against respiratory pathogens at young age. Moreover, the pace of adaptive-immune shifting may also account for the susceptibility to infectious diseases, although it may also be that effectiveness of vaccines and susceptibility to infectious diseases are not the same. Based on these notions, the major challenges ahead are to determine whether infants that are susceptible to (severe) infectious diseases are also those infants that respond less effectively to vaccination, or that these are two different processes. Following on this, it would be vital to determine whether the stage at which the adaptive immune system resides in at that moment can be used as a proxy to predict susceptibility to disease and lower effectiveness of vaccination. Together, these insights may aid in vaccination strategies to determine the ideal time for vaccination of neonates and infants and whether adjusting vaccine formulations to specifically boost innate-like adaptive responses may confer greater protection than current strategies. Thus, identification of factors that drive the shift from innate-like to an adaptive immune system that successfully generate long term memory cells should be a primary objective for further research. Moreover, future research should then define whether a rapid shifter is less prone to infectious disease and responds better to vaccination compared to infants who are slower shifters. Collectively, these new insights will help optimizing vaccination strategies to be able to better protect young infants against respiratory infectious diseases.

## Author contributions

DP – reviewed literature, conceptualized, wrote, and revised the manuscript. MB – reviewed literature, conceptualized and revised the manuscript. JW – reviewed literature, conceptualized, and revised the manuscript. All authors contributed to the article and approved the submitted version.

## Funding

This study was supported by the Dutch Ministry of Health, Welfare and Sport.

## Acknowledgments

We thank Willem Luytjes and Teun Guichelaar for critically reviewing the manuscript and useful discussions. Figures were created with BioRender.com.

## Conflict of interest

The authors declare that the research was conducted in the absence of any commercial or financial relationships that could be construed as a potential conflict of interest.

## Publisher’s note

All claims expressed in this article are solely those of the authors and do not necessarily represent those of their affiliated organizations, or those of the publisher, the editors and the reviewers. Any product that may be evaluated in this article, or claim that may be made by its manufacturer, is not guaranteed or endorsed by the publisher.
